# The Relationship between Fatty Acids and Different Depression-Related Brain Regions, and Their Potential Role as Biomarkers of Response to Antidepressants

**DOI:** 10.3390/nu9030298

**Published:** 2017-03-17

**Authors:** Maria Fernanda Fernandes, David M. Mutch, Francesco Leri

**Affiliations:** 1 Department of Psychology and Neuroscience, University of Guelph, Guelph, ON N1G 2W1, Canada; mariafer@uoguelph.ca; 2 Department of Human Health and Nutritional Sciences, University of Guelph, Guelph, ON N1G 2W1, Canada; dmutch@uoguelph.ca

**Keywords:** mental health, mental illness, diet, fatty acids, antidepressants, HPA-axis, hippocampus, striatum, prefrontal cortex

## Abstract

Depression is a complex disorder influenced by a variety of biological and environmental factors. Due to significant heterogeneity, there are remarkable differences in how patients respond to treatment. A primary objective of psychiatric research is to identify biological markers that could be used to better predict and enhance responses to antidepressant treatments. Diet impacts various aspects of health, including depression. The fatty acid composition of the Western diet, which has a high ratio of *n*-6:*n*-3 polyunsaturated fatty acids, is associated with increased incidence of depression. The brain is rich in lipids, and dietary fatty acids act within specific brain regions to regulate processes that impact emotional behavior. This manuscript reviews existing evidence demonstrating brain region-specific fatty acid profiles, and posits that specific fatty acids may serve as predictive biomarkers of response to antidepressants. Furthermore, increasing blood levels of certain fats, such as *n*-3s, via dietary intervention may serve as an adjunct to improve the efficacy of antidepressants. Notably, most of the existing research regarding fats and depression-related brain regions has focused on *n*-3s, as compared to *n*-6s, monounsaturated, and saturated fats. This review article will help guide future work investigating the relationships between fatty acids, brain regions, and antidepressant efficacy.

## 1. Introduction

Depression is one of the most common types of mental illness worldwide, affecting females twice as much as males [1]. This psychiatric disorder is of increased prevalence in Westernized countries and has significant personal and socioeconomic consequences [2]. Estimates from the World Health Organization (WHO) suggest that, by 2020, depression will be the second-leading cause of disease burden across the globe after heart disease [2]. Depression is a multifactorial disorder and both genetic and environmental factors, such as diet [3], are thought to play important roles in its etiology and treatment.

There is considerable interest to better understand how ingested nutrients impact the development and/or severity of mental illness. Diets which lack essential nutrients have adverse consequences on overall brain function and, consequently, mental health. In contrast, the intake of diets rich in whole grains, fish, poultry, fruits and/or vegetables, have been suggested to prevent depressive disorders (for review see [4,5,6,7]).

Diet impacts different physiological mechanisms that may, consequently, have a role modulating risk and development of depression. In recent years, an emerging field of research known as nutritional neuroscience has been focusing on the relationship between diet and depression [8]. Dietary fats represent a specific class of nutrients often associated with mental health. Specifically, saturated fats have been correlated with symptoms of depression in humans [9]. In contrast, dietary monounsaturated fats tend to be inversely associated with symptoms of depression in humans [10]. Similarly, dietary *n*-3 fats are most often associated with improvements in depression, although there is some inconsistency in the literature [11,12]. Finally, positive associations between *n*-6 fats and risk of suffering from severe depression in humans [10] have been reported. Together, this highlights the need to consider dietary fat composition when studying factors that influence the etiology and the treatment of depression. 

Dietary fat intake is reflected in the fatty acid composition of the brain, and some region-specific differences have been observed. This is particularly relevant to consider given that the pathophysiology of depression may be distributed across several brain regions, including the hippocampus, hypothalamic-pituitary-adrenal axis, prefrontal cortex and striatum. This suggests that unraveling the relationship between dietary fats and depression may need to consider specific regions of the brain.

Despite various advances in pharmacotherapy during the past decades, the successful treatment of depression remains challenging. Indeed, not all patients respond to treatments and considerable individual variability to existing treatments has been observed. Taking these issues into consideration, there is a need to better understand the various influences that contribute to the development of this disease, including genetics and lifestyle factors, to identify biomarkers and develop alternative pharmacotherapies. 

The present paper provides a timely review of the current state of knowledge regarding the roles of dietary fatty acids in brain regions implicated in depression. Furthermore, we provide compelling evidence that blood fatty acid profiles may serve as a potential predictive biomarker of response to antidepressant treatment.

## 2. Overview of Dietary Fats

Briefly, fatty acids are classified based on the number of double bonds (i.e., the degree of saturation/unsaturation) in the carbon chain into saturated (SFA; no double bonds), monounsaturated (MUFA; one double bond), or polyunsaturated (PUFA; two or more double bounds) [13]. PUFAs are further distinguished based on the position of the first double bond from the methyl terminal end into *n*-3 or *n*-6 PUFAs. While humans have the ability to, de novo, synthesize the majority of fatty acids, two PUFAs are essential to consume in the diet: alpha-linolenic acid (18:3*n*-3; ALA) and linoleic acid (18:2*n*-6; LA) [14]. These essential PUFAs can be endogenously converted (to a limited extent) through a series of desaturation and elongation steps into important longer-chain PUFAs. Specifically, ALA is metabolized into eicosapentaenoic acid (20:5*n*-3; EPA) and docosahexaenoic acid (22:6*n*-3; DHA), while LA is metabolized to arachidonic acid (20:4*n*-6; AA) [15].

In the modern diet, the primary sources of SFAs are eggs, fatty meats, and dairy products; while MUFAs are found in a variety of foods and oils, including olive and canola oil, avocados, hazelnuts, almonds, and pecans [16]. The major sources of *n*-3 PUFAs are fatty fish, green leafy vegetables, walnuts, and a variety of seeds (chia, flax, rape), while *n*-6 PUFAs are plentiful in nature and found in the seeds of most plants and oils, such as corn, sunflower, cottonseed, amongst others [15,16]. According to WHO recommendations, dietary fat should provide between 15%–30% of daily energy intake, where SFAs should contribute no more than 10% of daily energy intake and ~6%–10% should come from a balanced intake of *n*-3 and *n*-6 PUFAs [16]. While the optimal ratio of dietary *n*-6:*n*-3 is still debated, a ratio of 2–3:1 has been associated with a reduction in the incidence of inflammation, obesity and serious diseases, such as cancer [15,17]. However, the human diet has changed dramatically over the last century, with major changes occurring in both the type and overall quantity of fat consumed [18]. Indeed, the fatty acid composition of the typical Western diet is rich in saturated fats, with a high ratio of *n*-6:*n*-3 (15–20:1) [18,19]. This pattern of fatty acid intake is thought to correlate with the development of a number of metabolic and cognitive disorders, including depression [20]. While many studies suggest that a high dietary *n*-6:*n*-3 ratio is positively associated with risk of depression, there are also a few inconclusive or equivocal findings in the literature (for a review see [21]).

## 3. Role of Dietary Fatty Acids in the Whole Brain

Second to adipose tissue, the central nervous system has the greatest concentration of lipids in the body [22], primarily constituted by glycerophospholipids, glycerol ether lipids, cerebrosides, sulfatides, globosides, and gangliosides [22,23]. The fatty acid composition of the brain is extremely varied. The most abundant SFAs are palmitic (16:0) and stearic acid (18:0), while oleic acid (18:1*n*-9) represents the most common MUFA [14]. PUFAs, especially AA and DHA, are the dominant fatty acids in the brain. Specifically, the brain is highly enriched with DHA and has very low levels of LA, ALA, and EPA [14]. Furthermore, the fatty acid composition of different brain nuclei has been shown to vary, and this is thought to be related to the quantify of gray or white matter in nuclei [24]. For example, cerebral white matter has low levels of *n*-3 and *n*-6 PUFAs, but is rich in SFA and MUFA. In contrast, gray matter is highly enriched with *n*-3 PUFAs [25]. Of note, alterations in brain fatty acid composition, which are primarily driven by changes in dietary fat content, appear to be closely connected to impaired emotional behavior (for review see [26,27]).

Fatty acids are taken up from circulating blood into the brain through the blood-brain barrier (BBB). Of note, some fatty acids, such as SFAs and MUFAs, can be synthesized by *de novo* lipogenesis within the brain itself; however, brain PUFA content is primarily dependent on the diet [14]. The transport of fatty acids from blood into the brain is a complex mechanism due to the presence of tight junctions in the BBB and has been the subject of much debate (for review see [28]). Some investigators believe that fatty acids can move across membranes by simple diffusion, while others suggest a protein-mediated process [14]. Although this is an important area of investigation, further discussion of fatty acid transport mechanisms in the brain is beyond the scope of the current review.

Once fatty acids are within the brain, they can be subsequently converted into various metabolites, such as eicosanoids and endocannabinoids. Eicosanoids are hormone-like compounds that function locally, and are produced “on demand” from either AA or EPA/DHA released from membrane phospholipids. These molecules regulate a number of important physiological functions, including inflammation, and are necessary for normal brain function [29]. Eicosanoids derived from AA are generally considered pro-inflammatory, while those derived from EPA/DHA are characterized as anti-inflammatory [30]. Therefore, the high *n*-6:*n*-3 PUFA ratio in the Western diet favors the production of pro-inflammatory eicosanoids [29].

Another important class of fatty acid metabolites are the endocannabinoids, which are derived from AA. They act in the central nervous system to modulate synaptic plasticity, neurotransmitter release, and have a neuroprotective action [31]. Furthermore, AA is a precursor for two main endocannabinoids—arachidonoylethanolamide (AEA or anandamide) and 2-arachidonoylglycerol (2-AG). While higher levels of AA are associated with increased production of endocannabinoids [32], it remains unclear if brain endocannabinoid production is modified by dietary fat intake. For example, mice fed a soy diet rich in LA, which can be converted into AA endogenously, showed a trend towards reduced 2-AG levels in the brain, instead of the anticipated increase [33]. Similar findings were also observed in rats fed an AA-enriched diet for one week [34] and piglets supplemented with soybean and sunflower oils (which are enriched in *n*-6 PUFAs) [35].

## 4. Dietary Fat Regulation of Brain Function and Links to Depression

### 4.1. Saturated Fatty Acids and the Whole Brain 

There are several reports indicating that the neurochemistry and function of the brain can be influenced by dietary SFAs. Of note, these fatty acids have been shown to impair a number of brain circuits implicated in the regulation of mood [36], such as neuroinflammation and feeding behavior.

Evidence suggests that SFAs induce neuroinflammation by stimulating the release of pro-inflammatory cytokines and inducing apoptosis in astrocytes, the supporting cells of the central nervous system [37]. At the cellular level, treating an immortalized neuronal cell line (mHypoE-44) with palmitic acid (16:0) induced over-expression of the orexigenic neuropeptide-Y (NPY), suggesting that SFAs may play a role in the regulation of food intake [38]. Studies exploring the molecular mechanisms by which SFAs impact the rodent brain to affect emotional behavior demonstrated that the intake of these fats can cause impairments in the activity of the brain dopamine system [36,39,40]. Additionally, the intake of a moderately high-fat diet (21% kcal total energy from fat, with ~62% of the fat comprised of SFAs) reduced brain-derived neurotrophic factor (BDNF) levels in rodents [41]. This is important given that reduced BDNF levels in the human brain has been associated with depression, while treatment with an anti-depressant increased BDNF and improved symptoms of depression [42]. Consistent with the previously mentioned rodent study, both a short (1–3 weeks) [43] and prolonged (6 weeks) intake of a high-fat diet (~60% kcal total energy from fat - containing high SFA content) induced depression-like behavior in rodents, as reflected by a reduced immobility time in the well-validated forced-swim test [40]. Similarly, data from a human cross-sectional study reported a significant positive correlation between depressive symptoms and serum levels of palmitic acid [9].

The connection between SFA consumption and emotional behavior requires further study. However, existing data suggests that limiting the intake of high SFA foods may prevent neuroinflammation, obesity, as well as mood disturbances and depression.

### 4.2. Monounsaturated Fatty Acids and the Whole Brain 

The intake of MUFAs has been suggested to improve overall brain function. For example, Sartorius and collaborators observed that an eight-week exposure to a MUFA-enriched diet (63% kcal total energy) improved insulin signaling in the mouse brain [44]. This is notable given that brain insulin resistance promotes depression-like behavior in rodents [45]. Furthermore, low MUFA levels have been associated with the development of Alzheimer’s disease, as suggested by findings that MUFA levels, especially oleic acid, are reduced in the brain of Alzheimer’s patients [13]. Importantly, depression is commonly observed in Alzheimer’s patients [46].

Interestingly, high MUFA intake has been inversely associated with depression-like behavior. Hryhorczuk and collaborators observed that the intake of a diet high in olive oil (50% kcal total energy) for 8–9 weeks protected the integrity of the brain dopamine system in rodents, thereby reducing the risk of depression-like behavior [36]. Furthermore, the daily administration (5 mg/kg/day) of oleamide, a derivative of oleic acid, for two weeks significantly elevated hedonic responses in rats, as indicated by increased consumption in a sucrose consumption test [47]. Oleamide administration also suppressed depression-like behavior, assessed by the forced-swim test [48]. Consistent with the notion that dietary fats can influence brain lipid composition, the long-term (11 weeks) consumption of a MUFA-enriched diet (fat from olive oil) significantly increased total MUFA levels in the mouse brain and concomitantly reduced SFA levels [49]. Notably, a high MUFA:SFA ratio has been shown to increase brain membrane fluidity [49], which can facilitate neurotransmitter signal transduction and improve mental health. In agreement with these pre-clinical findings, prolonged intake (between 6 and 10 years) of a MUFA-enriched diet in humans reduces risk of depression [10,50]. Furthermore, an epidemiological study suggested that the consumption (~4 years) of a Mediterranean diet, which is high in MUFA-rich olive oil, had a potentially protective effect in depressive disorders. The authors observed that individuals who adhered to the Mediterranean diet had a lower incidence of depression, although they could not explain the molecular mechanisms by which the prolonged intake of a MUFA-rich diet produced this effect [51].

### 4.3. Polyunsaturated Fatty Acids and the Whole Brain 

PUFAs have several important roles in the central nervous system, such as regulation of food intake and glucose homeostasis. In addition, these fatty acids play a crucial role regulating apoptosis, neuroinflammation, neurotransmission, and emotional behavior (for review see [14]). 

#### 4.3.1. Dietary *n*-3 Polyunsaturated Fatty Acids

There is considerable interest to better understand the role of *n*-3 PUFAs in the central nervous system given that these fatty acids are highly enriched in the brain and are critical for brain development, cognition and behavioral functions. Moreover, these fatty acids regulate a number of neurotransmitter functions, including responsivity, signal transduction, and phospholipid turnover [52]. *n*-3 PUFAs have also been associated with psychiatric disorders and are involved in cognitive development, prevention of neuronal death, and the prevent/attenuation of neuroinflammation [13].

In regards to emotional behavior, dietary *n*-3 PUFA deficiency has been shown to alter the regulation of both dopaminergic and serotonergic neurotransmissions in both preclinical and clinical studies [11,53]. Experimental findings in rodents demonstrated that a deficiency in dietary *n*-3 PUFA induced a depression-like phenotype in the forced-swim test, while increased *n*-3 PUFA intake had the opposite effect and ameliorated the depressed phenotype. This suggests that *n*-3 PUFAs may have “antidepressant” activity [12]. However, there is inconsistency in the literature about the relationship between *n*-3 PUFAs and anhedonia-like behavior. For example, it was previously suggested that the intake of an ALA-deficient diet induced anhedonia in mice, as reflected by reduced preference for sucrose in a sucrose preference test [54]. But other studies demonstrated that rats maintained on the same ALA-deficient diet for several generations did not display an anhedonic phenotype on the sucrose preference test [53]. Furthermore, chronic treatment with *n*-3 PUFA (180 mg of EPA and 120 mg of DHA/1000 mg of fish oil, administered at a dose of 500 mg/kg) failed to reverse anhedonia in rats exposed to chronic mild stress [55]. In this latter work, the authors measured the consumption of sweet food (a mix of wheat pellets, cornstarch and sucrose—Kellogg’s^®^ Froot Loops^®^ cereal) as an index of anhedonia rather than the classic sucrose preference test. It is also important to mention that other rodent studies demonstrated that *n*-3 PUFAs are essential for the integrity of the BBB [56], and for a proper blood supply to the brain [57,58]. This is relevant given that some clinically depressed patients suffer from impaired brain blood flow [59,60].

Studies examining the link between *n*-3 fatty acids and depression in humans have produced mixed results. There is evidence that the intake of these fatty acids may lower depressive symptoms in adults [11], and that blood levels of *n*-3 PUFAs are inversely associated with depression [61,62]. However, some reports failed to find an association between *n*-3 PUFA intake and depression [9,63,64], while others suggested that patients with unipolar and postpartum depression have lower *n*-3 PUFA levels [65,66]. The inconsistency in the literature regarding the effects of *n*-3 PUFAs may stem from variability in experimental design, differences in lifestyle behaviors between study populations, and/or the duration of exposure to these fatty acids. 

The molecular mechanisms underlying the action of *n*-3 PUFAs in the brain needs further clarification. These PUFAs influence the activity of many signaling pathways by regulating BDNF and insulin-like growth factor 1 (IGF1). Of note, IGF1 supports neurotransmitter synthesis and release, and synaptic plasticity, amongst other functions [67]. BDNF and IGF1 can subsequently activate signaling cascades, such as the mitogen-activated protein kinase (MAPK) and calcium/calmodulin-dependent protein kinase II (CaMK II), involved in synaptic transmission and long-term potentiation (LTP); mechanisms previously associated with emotion, learning and memory [67]. Taken together, these findings suggest that *n*-3 PUFA have important roles in brain development and function, and may be beneficial to attenuate symptoms of depression. 

#### 4.3.2. Dietary *n*-6 Polyunsaturated Fatty Acids 

Similar to *n*-3 PUFAs, *n*-6 PUFAs also play a major role in the brain by influencing growth, development and function [68]. Previous studies examining tissue fatty acid composition have demonstrated that dietary *n*-6 PUFAs affect brain lipid content. Dietary *n*-6 deficiency decreased *n*-6 PUFA levels in the brain membranes of piglets, and this decrease was associated with reduced concentrations of both dopamine and serotonin [69]. This suggests that *n*-6 PUFA may play a role in controlling the synthesis of these neurotransmitters and provides strong evidence for the role of *n*-6 PUFAs in normal brain function.

Notably, *n*-6 PUFAs have been shown to increase the levels of BDNF in the brain [70,71], although the molecular mechanisms involved in this action remain unclear. The available data is limited and inconsistent when it comes to understanding the link between *n*-6 PUFA and depression. Pre-clinical studies reported increased levels of *n*-6 PUFA in the brain of the Flinders Sensitive Line rat, an animal model of depression [72]. In addition, oral administration of LA (1 mg/kg/day) for 10 days failed to reverse the depressed phenotype of a mouse model of depression, while the administration of a derivative of this fatty acid, 8-[2-(2-pentyl-cyclopropylmethyl)-cyclopropyl]-octanoic acid (DCP-LA; 0.5–5 mg/kg), improved their depression-like behavior in the forced-swim test in a dose-dependent manner. Kanno and colleagues suggested that, when administered orally, LA is rapidly metabolized and has poor penetration into the brain. In contrast, DCP-LA is more stable and consequently has greater bioactivity in the central nervous system [73]. Similar to pre-clinical findings, the evidence in humans is equally inconsistent. For example, it was previously reported that serum *n*-6 PUFA levels are inversely correlated with depression [9], but Wolfe and colleagues observed a positive association between dietary *n*-6 PUFA intake (and corresponding blood levels) and risk of severe depression [10]. Furthermore, data from a cross-sectional study in pregnant Brazilian women found that high serum levels of *n*-6 PUFAs were associated with a greater likelihood of depression [74]. 

The findings described above suggest that *n*-6 PUFAs play an important role in brain function. In addition, despite the inconsistency in the literature, *n*-6 PUFAs seem to be associated with depression (for review see [21]). A better understanding of *n*-6 PUFAs in the brain, and how this may be modulated through the diet, represents an important avenue for further investigation.

## 5. Role of Dietary Fatty Acids in Specific Brain Regions Involved in Depression

A major challenge in defining the molecular basis of depression is that a number of different brain areas (i.e., prefrontal cortex, striatum, hippocampus, and hypothalamic-pituitary axis) and circuits are involved in the etiology and treatment of depression [75]. This suggests that the pathophysiology of depression may be distributed across the brain, and highlights the need to investigate possible differential fatty acid functions in different regions of the brain. Thus, the following sections provide an overview of the existing knowledge regarding fatty acids in specific brain regions and relationships with depression.

### 5.1. Dietary Fats, Depression, and the Hippocampus

The hippocampus is one of the primary subcortical limbic brain regions implicated in depression. Structural and functional abnormalities leading to dysfunction of this region have been associated with depression. For example, patients with depression have lower hippocampal volume [76] and are more prone to relapse after treatment, compared to those with larger hippocampal volumes [77]. It remains unclear why depression is associated with volume reduction of the hippocampus, but neuronal loss or inhibition of neurogenesis may play a role [75]. Interestingly, hippocampal fatty acid content has been shown to vary with depression and depression-like behaviors, and can be altered by dietary manipulation. As such, examining the role of fatty acids in the hippocampus, and how they may influence depression represents an interesting area of investigation.

*Saturated Fats:* The hippocampus is enriched with SFAs, which has been shown to disrupt hippocampal-related functions in rodents [24], possibly via their pro-inflammatory and pro-apoptotic actions [37]. Furthermore, maternal consumption of an SFA-rich high-fat diet (60% kcal total energy) for nine weeks in mice induced alterations in hippocampal gene expression in the serotonergic system and BDNF in adult offspring [78]. To date, there is no evidence that changes in SFA levels in the hippocampus can influence depression in humans.

*Monounsaturated Fats:* To the best of our knowledge, there is limited data regarding the role of MUFAs in the hippocampus and their relation to depression. Specifically, there is no pre-clinical evidence available. However, data collected in healthy adults who consumed a diet high in MUFA-rich olive oil (i.e., Mediterranean diet) for ~4.4 years demonstrated that this diet modulated hippocampal-related function, such as emotional behavior, and that this was associated with a reduced incidence of depressive symptoms [79]. 

*n*-*3 PUFAs:* Some pre-clinical studies have proposed that *n*-3 PUFAs act in the hippocampus to prevent or reduce the incidence of depression. Rats fed an EPA-enriched diet for six weeks showed increased hippocampal concentrations of dopamine and serotonin [80], suggesting that this *n*-3 PUFA can prevent depression-like behavior. Consistent with this, a maternal deficiency of dietary *n*-3 PUFA impaired neurogenesis and decreased levels of serotonin and norepinephrine in the hippocampus of neonatal rat offspring [81]. Despite the existence of this pre-clinical evidence, Hamazaki and colleagues did not detect robust changes in *n*-3 PUFA levels in the post mortem hippocampus of humans with diagnosed mood disorders. Instead, the authors reported changes in *n*-6 PUFA content, suggesting that hippocampal *n*-6 PUFA, rather than *n*-3 PUFA, may be associated with depression [82].

*n*-*6 PUFAs:* There is limited and inconsistent data regarding the effects of dietary *n*-6 PUFAs in the hippocampus and the relationship with depression-like behavior. A study conducted in mice showed that the LA derivative DCP-LA improved depression-like behavior in animals exposed to restraint stress and tested in the forced-swim test, without affecting hippocampal serotonin neurotransmission [73]. However, another study showed that rats fed an *n*-6 PUFA-enriched diet containing safflower oil for eight weeks had reduced serotonin levels and neurotransmission in the hippocampus [83]. It is notable that impairments in serotonergic neurotransmission are thought to increase vulnerability to depression in both rodents and humans. A similar inconsistency also exists in human studies. For example, Jacka and collaborators observed that prolonged intake of a Western diet, which is abundant in *n*-6 PUFA, increased the incidence of depression and is associated with smaller hippocampal volume in older adults [84]. However, Hamazaki and colleagues revealed that the post mortem analysis of the hippocampus in human adults with diagnosed mood disorders showed reductions in *n*-6 PUFA content [82].

### 5.2. Dietary Fats, Depression, and the Hypothalamic-Pituitary-Adrenal (HPA) Axis 

There is evidence that abnormalities in the HPA-axis contribute to the pathophysiology of depression [85], possibly via perturbations in fatty acid metabolism [86]. However, the direct contribution of different fatty acids on this region has been poorly explored and requires further investigation.

*Saturated Fats:* Sharma and Fulton demonstrated that mice exposed to a high SFA diet for 12 weeks showed a depression-like phenotype in the forced-swim test, accompanied by elevated cortisol levels and HPA activity. This suggests a potential role for cortisol, a glucocorticoid hormone produced upon HPA-axis activation, in depression-like behavior [40]. In addition, maternal intake of a high fat that was moderately rich in SFAs (32% kcal total energy) dysregulates hypothalamic serotonin turnover and induces depression-like behaviors in macaque offspring [87]. Finally, there is evidence that dietary SFAs induce HPA disturbances (e.g., hyperactivity) and increase vulnerability to depression in humans [88].

*Monounsaturated Fats*: To date, there is no evidence that MUFA levels or changes in dietary MUFA intake affect the HPA axis to influence depression-like behaviors.

*n*-*3 PUFAs:* The link between the actions of *n*-3 PUFAs on the HPA axis and depression remains unclear. Nevertheless, maternal dietary *n*-3 PUFA deficiency in rats was shown to provoke HPA hyperactivity (as observed by elevated cortisol levels) and cause depression-like behaviors (assessed by the forced-swim test) in offspring [89]. Consistent with this, studies in humans have shown an inverse association between cortisol levels and blood concentrations of EPA and DHA in depressed individuals [86]. In addition, an eight-week dietary supplementation with EPA decreased HPA-axis activity (as observed by reduced serum cortisol) in patients diagnosed with depression and improved depressive symptoms in these same patients [90]. Of note, corticosteroids are known to down-regulate the transcription of the serotonin 1A receptor gene in the limbic system [91], suggesting that manipulations that decrease cortisol levels may affect serotonin neurotransmission and ameliorate depressive symptoms. 

*n*-*6 PUFAs:* Kanno and collaborators observed that oral administration of LA (1 mg/kg/day) for 10 days failed to affect hypothalamic serotonin neurotransmission and did not influence depression-like behavior in mice tested in the forced-swim test [73]. In contrast, another study demonstrated that maternal exposure to a *n*-6 PUFA-enriched diet (43% kcal total energy) in mice induced behavioral despair in the forced-swim test, accompanied by increased protein kinase C (PKC) signaling in the hypothalamus of the offspring [92]. Of note, increased PKC activity in this region is associated with depression and other psychiatric disorders [93]. To the best of our knowledge, there is no evidence that *n*-6 PUFA levels, or changes in *n*-6 PUFA dietary intake, affect the HPA axis to influence depression in humans.

### 5.3. Dietary Fats, Depression, and the Prefrontal Cortex (PFC) 

The PFC is well known for its role in reward, executive functions and regulation of emotional behaviors [94]. The PFC has been the subject of much investigation in the pathophysiology of depression, as decreased metabolism and blood flow in this brain site is commonly observed in depressed individuals [75]. Dietary fats differently impact this area to affect depression, similar to what was observed in other brain nuclei.

*Saturated Fats:* Few studies have addressed the specific roles of SFAs in this region and their association with depression. A study by Guida and colleagues recently demonstrated that chronic administration of the endocannabinoid palmitoylethanolamide (PEA; 10 mg/kg, i.p. for 15 days), which is derived from palmitic acid, alleviated a depression-like phenotype in mice, partially by acting on the PFC to promote glutamatergic synapse homeostasis [95]. More specifically, the authors observed that systemic treatment with PEA significantly reduced the time of immobility in the tail suspension test [95]. However, post mortem studies investigating the fatty acid composition in the PFC of patients with depression did not find a significant difference in SFA levels between depressed and control groups [96].

*Monounsaturated Fats*: To date, there is no evidence that MUFA levels affect the PFC to influence depression-like behaviors. However, a study by Lalovic and colleagues noted that PFC MUFA levels are not different in post-mortem brain tissue from patients with depression, as compared to controls [96].

*n*-*3 PUFAs:* Delion and collaborators demonstrated that a deficiency in dietary *n*-3 PUFA alters the membrane phospholipid composition of the PFC. Specifically, the authors observed lower levels of *n*-3 PUFAs in the PFC of rats chronically exposed to an ALA-deficient diet. Moreover, *n*-3 PUFA deficient animals had reduced levels of endogenous dopamine and altered serotonin neurotransmission in the PFC [97], which may indicate that these animals are more susceptible to develop a depression-like phenotype. Consistent with these findings, Lafourcade and colleagues demonstrated that a lifelong dietary imbalance in the ratio of *n*-6:*n*-3 PUFAs changed fatty acid composition in the PFC and triggered depressive-like symptoms in mice subjected to the forced-swim test [98]. Further evidence of the importance of EPA on neurotransmission was demonstrated by increased levels of the serotonin-precursor tryptophan in the rat PFC after seven weeks consuming *n*-3 PUFA [80]; however, this study did not observe changes in PFC dopamine levels in rats fed an *n*-3 PUFA-deficient diet [80]. 

In addition to inducing alterations in PFC dopamine and serotonin levels, *n*-3 PUFAs also regulates PFC endocannabinoid signaling. It was previously demonstrated that an *n*-3 PUFA deficiency impaired endocannabinoid synaptic transmission in the PFC, an event that may contribute to the etiology of psychiatric disorders [98]. Furthermore, a life-long deficiency in *n*-3 PUFA intake in mice decreased brain levels of DHA, disrupted endocannabinoid neurotransmission in the PFC, and caused depression-like behavior in the forced-swim test [99].

DHA is the primary *n*-3 PUFA in the adult human PFC, accounting for ~15% of total fatty acid. Although available data are not consistent, some post-mortem studies have reported profound deficits of DHA in the PFC of depressed patients, an effect that may increase vulnerability to depression by increasing neuronal atrophy (for review see [100]). However, findings from Lalovic and collaborators did not show any significant alterations in *n*-3 levels in the PFC of depressed patients [96].

*n*-*6 PUFAs:* The involvement of *n*-6 PUFAs in depression is suggested by the observation that increasing total brain levels of *n*-6 PUFA-derived endocannabinoids, such as AEA, impaired serotonin neurotransmission in the PFC, and induced a depression-like phenotype in mice [101]. Similar to what was observed for *n*-3s in this region, data are also inconsistent for *n*-6s in the PFC. Studies conducted in post-mortem human PFC samples found reduced levels of DHA and elevated AA:DHA ratio in membrane phospholipids of patients diagnosed with depression [102,103]. However, another study also conducted in post mortem PFC samples of depressed men who committed suicide did not find alterations in *n*-6 levels in the PFC [96].

### 5.4. Dietary Fats, Depression, and the Striatum 

The striatum is a subcortical region of the forebrain that has been a focus in studies exploring depression, primarily due to its role in reward and motivation [75]. Importantly, it is believed that depression-related anhedonia reflects disturbances in the mesolimbic dopamine system, which has been linked to several aspects of reward processing [104]. Of note, some studies have demonstrated that fatty acids may act on striatal regions to influence depression-like behaviors. Examining the role of different fats in the striatum of humans and how this may contribute to depression represents an important line of investigation.

*Saturated Fats:* The role of SFA in the striatum and its association with depression was demonstrated in a study conducted in adult male mice fed a SFA-rich diet (58% kcal total energy) for 12 weeks. It was observed that chronic consumption of SFA induced depression-like behavior in the forced-swim test, accompanied by increased striatal levels of BDNF [40]. Furthermore, an eight-week exposure to a high-SFA diet (from beef tallow) increased post-synaptic serotonin 2A receptor binding in the dorsal striatum of rats [83], a mechanism that has been previously linked to depression [105]. However, a study investigating fatty acids in post mortem brain tissue observed no differences in SFA in the striatum (caudate regions) of patients with psychiatric disorders [106].

*Monounsaturated Fats:* To date, there is no available clinical or pre-clinical data on the effects of dietary MUFAs in the striatum to influence depression. 

*n*-*3 PUFAs:* Interestingly, the prolonged consumption of an *n*-3 PUFA deficient diet impaired dopamine signaling [107] and decreased BDNF levels in the striatum of rodents [108], suggesting an increased vulnerability to depression-like behaviors. However, Yao and collaborators failed to find differences in *n*-3 PUFA levels in the post mortem striatum of patients with psychiatric disorders, other than schizophrenia [106].

*n*-*6 PUFAs:* Although available evidence is limited regarding the importance of dietary *n*-6 PUFAs in the striatum in depression, dietary intake of these fatty acids act on this region to induce a depression-like phenotype, as suggested by data showing that rats fed a high *n*-6 PUFA diet (i.e., enriched with safflower oil) for eight weeks have reduced striatal serotonin levels [83]. However, there were no differences in *n*-6 levels in the striatum of patients with depression, as compared to normal healthy controls [106].

## 6. Blood Fatty Acids as Predictors of Response to Antidepressant Treatments

The heterogeneity of depressive symptoms, response to treatment, and underlying pathophysiology make the development of efficacious “personalized” treatments challenging. 

Currently there are many different drug options available to treat depression. However, it is impossible to predict which of these treatments will work best for a given patient. Consequently, clinicians often explore different drugs and/or doses before finding a treatment that works efficaciously and has minimal side-effects for the patient. Therefore, the identification of biomarkers that can be used to characterize treatment response is critical to the development of improved therapies and appropriate treatment options for different depressed individuals, as well as for improving our understanding of the pathogenesis of depression [109].

**Figure 1:**
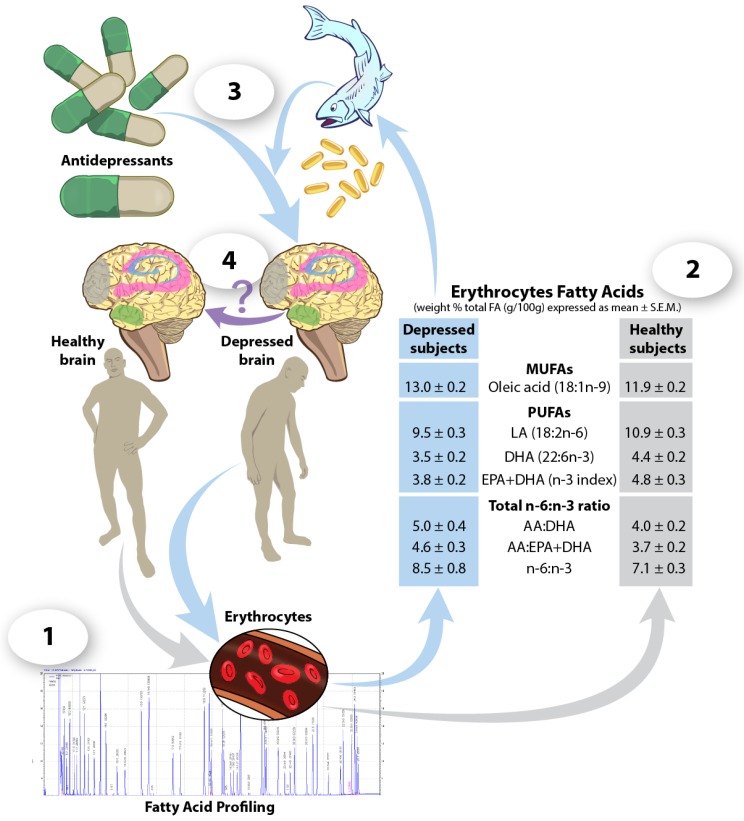
Dietary fats as a potential adjunct to antidepressant medication. (**1**) Profiling erythrocyte fatty acids can be used to distinguish healthy from depressed individuals, and identify any deficiencies in specific fats. (**2**) For example, there is evidence that depressed patients show significant deficits in blood *n*-3 PUFA levels as compared to healthy controls [112]. (**3**) Increased intake of fatty acids, such as *n*-3 PUFAs, via the consumption of fatty fish or dietary supplements may serve as an adjunct therapy to improve efficacy of antidepressant treatment. (**4**) It is currently unknown if using dietary fats in combination with antidepressant treatments modifies the fatty acid composition of the hippocampus (pink), HPA-axis (green), PFC (grey), and striatum (blue)of a depressed individual’s brain to resemble that of a healthy individual.

This review highlighted significant associations between fatty acids and mental health. Consequently, this suggests that a person’s blood fatty acid profile may serve as a potential biomarker of predisposition for depression and/or treatment responsiveness (Figure 1). In support of this idea, significant differences in the plasma lipid profile of young adults (27–33 years of age) with major depressive disorder compared to age-matched healthy controls have been observed. Specifically, plasma levels of specific phospholipids, lysophospholipids and triacylglycerols were increased in depressed individuals and positively associated with severity of depression [110]. Further, plasma levels of total free fatty acids and glycerophospholipids were reduced in these patients and inversely associated with the severity of depression. Collectively, this suggests that plasma lipid profiles could be used to diagnose individuals with this mental disorder [110]. In addition, the evidence presented in the current review suggests that blood levels of specific fatty acids (e.g., *n*-3 PUFAs) and fatty acid metabolites (e.g., endocannabinoids) may serve as promising markers to assess a person’s risk and vulnerability for depression. For example, DHA levels in erythrocytes of both Caucasian children and adolescents (9–18 years old) [111] and adults (31–37 years old) were found to be lower in those diagnosed with depression, as compared to healthy controls [112] (Figure 1). Likewise, McNamara and collaborators observed reduced erythrocyte levels of *n*-3 PUFAs in adolescents (9–20 years old) with varying risk for developing mood disorders [113]. 

Interestingly, there are studies suggesting that PUFA metabolites, such as endocannabinoids, may be a useful and alternative biomarker for depression. Hill and collaborators observed reduced serum levels of AEA and 2-AG in adult female patients diagnosed with depression [114,115]. Of note, the authors also reported that serum 2-AG content was inversely correlated with the duration of the depressive episode [115]. Based on this evidence, endocannabinoids may also serve as possible biomarkers for depression. These findings suggest that blood lipids (e.g., fatty acids, complex lipids, or specific fatty acid metabolites) could differentiate individuals who are depressed from those who are not. However, blood lipids are influenced by a number of factors, including diet, physical activity, smoking, and genetics [116]. As such, further investigation is necessary to determine if these factors compromise our ability to use blood lipids as a biomarker to predict treatment response.

Biomarkers can also be used in therapeutic drug development and improvement of treatment response. In this context, *n*-3 PUFAs may serve as an adjunct therapy to improve response to some antidepressants (Figure 1). For example, Jazayeri and collaborators observed that an eight-week treatment with either the selective serotonin reuptake inhibitor fluoxetine (20 mg) or EPA (500 mg per day) appeared to be equally effective in controlling depression in adults (20–59 years old) diagnosed with depression (based on a score ≥ 15 in the 17-item Hamilton Depression Rating Scale (HDRS)). However, the combination of fluoxetine and EPA was even more effective at reducing depressive symptoms (~81% decrease in baseline HDRS) than either of them alone (≥50%–56% decrease in baseline HDRS) [117]. This suggests that there may be greater benefits when fluoxetine is combined with a daily *n*-3 PUFA supplement. Similarly, Gertsik and colleagues demonstrated that a 9-week treatment of citalopram (20 mg) supplemented with a daily dose of *n*-3 PUFA consisting of 900 mg of EPA + 200 mg DHA resulted in a greater efficacy (~60% decrease in baseline HDRS) compared to citalopram alone (~20% decrease in baseline HDRS) [118]. Other studies have reported similar findings [119,120,121,122,123], supporting the hypothesis that some fatty acids, such as *n*-3 PUFAs, can be used as a beneficial and safe adjunct to enhance the therapeutic effects of antidepressants in depressed patients. This suggests that measuring blood *n*-3 PUFA levels may serve as a valuable biomarker to predict high and low responders to antidepressant treatments. Moreover, increasing *n*-3 PUFA levels in low responders through foods and/or dietary supplements may improve the efficacy of pharmacotherapies (Figure 1). However, it remains unclear if the use of dietary fats as an adjunct to pharmacotherapy causes a remodeling of fatty acid composition in specific brain regions to more closely resemble that of a healthy individual.

## 7. Future Perspectives

Future studies are necessary to address important gaps in knowledge regarding the role of dietary fats as potential mediators of depression and, most importantly, to validate the hypothesis that they can be used as biomarkers of antidepressant response. It would be interesting and relevant to conduct further research in larger populations of males and females to reproduce the results obtained with *n*-3 PUFA supplementation using different classes of antidepressants and different treatment periods. It would also be valuable to determine if different *n*-3 PUFA (ALA vs. EPA. vs. DHA) have similar effects or not. Moreover, the evidence that blood levels of some fatty acids and their metabolites are related to depression is predominantly correlational at this time, while brain fatty acids are not typically investigated. Future research should strive to explore brain region-specific fatty acid profiles in healthy individuals and those at risk and/or diagnosed with depression. Using non-invasive medical imaging technologies, such as positron emission tomography and single photon emission computed tomography, one could quantitatively image regional fatty acid distribution in the human brain and characterize region-specific brain fatty acid profiles of depressed and non-depressed individuals. Some of these techniques have already been used successfully in humans to study fatty acid incorporation from plasma into the brain [124], region-specific brain fatty acid metabolism [125], and to detect brain fatty acid levels in neurodegenerative diseases [126].

## 8. Conclusions

Accumulating evidence from clinical and pre-clinical studies suggests that fatty acids and some of their metabolites act within specific brain regions to regulate a number of processes, such as neurotransmission and signaling pathways, which ultimately affects emotional behavior. However, the underlying neurobiological mechanisms, and whether the relationship between fatty acids and depression stems from a brain region-specific remodeling of fatty acid profiles rather than a global whole-brain remodeling remains unclear. Finally, while circulating lipids can distinguish individuals who are depressed from those who are not, their use as biomarkers to predict response to pharmacotherapy requires further exploration. However, there is encouraging evidence to suggest that blood lipid profiles could be used to predict a person’s response to pharmacotherapy and that changing and/or supplementing dietary fat intake could help to improve the efficacy of antidepressant treatments.
